# Impact of excessive gestational weight gain on exclusive breastfeeding among women with Type 1 and Type 2 diabetes and obesity

**DOI:** 10.1371/journal.pone.0277599

**Published:** 2022-11-17

**Authors:** Leandro Cordero, Michael R. Stenger, Mark B. Landon, Craig A. Nankervis

**Affiliations:** 1 Department of Pediatrics, College of Medicine, The Ohio State University Wexner Medical Center, Columbus, OH, United States of America; 2 Department of Obstetrics and Gynecology, College of Medicine, The Ohio State University Wexner Medical Center, Columbus, OH, United States of America; University of Cambridge, UNITED KINGDOM

## Abstract

**Background:**

Pregestational diabetes, obesity and gestational weight gain (GWG) are associated with adverse perinatal outcomes, however, the influence of *excessive* GWG on lactation at discharge is less known. Our aim is to evaluate the impact of *excessive* GWG using the LifeCycle project guidelines on exclusive breastfeeding (EBF) and any BF rates at discharge among 171 women with Type 1 and 294 Type 2 diabetes and obesity who intended to BF.

**Methods and findings:**

Retrospective cohort study. Obesity was defined by BMI (kg/m^2^) as grade 1 (30–34.9), grade 2 (35–39.9) or grade 3 (≥40). GWG was categorized as *adequate*, *inadequate or excessive* according to the 2019 LifeCycle Project guidelines. Women with Type 1 were younger (30 vs 33y), primiparous (51 vs 32%), delivered earlier (37 vs 38w) than women with Type 2 andwere different in grade 1 (40 vs 26%), grade 3 obesity (19 vs 49%) and median GWG (15 vs 11kg). Of all 465 women with Type 1 and Type 2 combined, 365 (78%) who had *excessive* GWG and 100 (22%) who had non-*excessive* GWG showed similar EBF (27 vs 25%) and any BF (72 vs 72%) rates. Regression analysis showed that after adjusting for potential confounders *excessive* GWG was not a predictor of EBF or any BF at discharge.

**Conclusion:**

Type 1 and Type 2 diabetes, obesity and excessive GWG are associated with low EBF, however, *excessive* GWG is not an independent predictor of low EBF or any BF at discharge.

## 1. Background

Lactation provides short- and long-term health benefits to mothers and infants following healthy as well as high risk pregnancies [1–4]. More specifically, exclusive breastfeeding (EBF) during birth hospitalization and the first postpartum year have been designated desirable goals [5,6]. Comorbidities such as gestational or pregestational diabetes mellitus and maternal obesity have been independently recognized as significant obstacles to BF initiation and duration [7–11].

Among healthy women gestational weight gain (GWG) has received attention from investigators and clinicians because it is a potentially modifiable factor which may impact pregnancy outcomes [12,13]. The Institute of Medicine (IOM) 2009 guidelines recommended GWG to be 5-9kg (10–20 lbs) for all women with BMI ≥ 30kg/m^2^ [12,13]. Since then, several investigations have focused on the impact of *adequate*, *inadequate* or *excessive* GWG on maternal and neonatal outcomes [12,13]. GWG above the IOM target range has been associated with an increased risk of gestational diabetes, pregnancy-related hypertension, macrosomia, large for gestational age infants and cesarean delivery [12,13]. To date, few publications have addressed the impact of *excessive* GWG on EBF or BF duration [14–17].

One limitation of the 2009 guidelines was the failure to provide specific GWG recommendations for women among different obesity classes as well as for women with diabetes [13]. In 2019, the LifeCycle project proposed specific GWG guidelines for women with grade 1 obesity (BMI 30–34.9kg/m^2^), grade 2 (BMI 35–39.9kg/m^2^) and grade 3 (BMI ≥ 40kg/m^2^) [18]. However, studies of associations of *excessive* GWG with EBF and BF initiation using the new guidelines are not available.

### 1.1 Objective

Our aim was to evaluate the impact of *excessive* GWG using the LifeCycle project guidelines on EBF and any BF rates at discharge among 171 women with Type 1 and 294 women with Type 2 diabetes and obesity who intended to BF.

## 2. Methods and findings

The Ohio State University Biomedical Science IRB approved on 5/02/2022 the continuation of the study #2010H0198 with waivers of informed consent and HIPAA research authorization. All methods were performed in accordance with the relevant guidelines and regulations of the declaration of Helsinki. Electronic maternal and neonatal records (2013–20) were reviewed. Some information obtained from women who delivered between 2013 and 2018 was used in previous investigations [7–9]. Type 1 and Type 2 pregestational diabetes, chronic hypertension (CHTN) and preeclampsia were diagnosed and treated in accordance with established guidelines [19–21]. GWG was calculated using weight at delivery minus pregestational weight measured during the first trimester or recalled at the time of delivery [12]. In the present study, women were categorized by prepregnancy BMI as normal (18.5–24.9 kg/m^)^, overweight (25–29.9 kg/m^2^), obese grade 1 (30–34.9 kg/m^2^), obese grade 2 (35–39.9 kg/m^2^) or obese grade 3 (≥ 40 kg/m^2^) [18]. According to LifeCycle guidelines, GWG was categorized as a*dequate* (within guidelines), *inadequate* (below) *o*r *excessive* (above) [13,18].

All women delivered singleton infants at ≥ 34 weeks gestation; pregnancies affected by major fetal malformations were not included in the study. Health insurance type was used as a proxy for socioeconomic status [22]. Upon arrival to labor and delivery, each woman described her past BF experience and her intention to exclusively or partially BF. In our institution, maternity practices include BF within 1 hour of delivery, no formula supplementation unless indicated, rooming in, on demand BF, full-time lactation consultants and post discharge BF support [8,9]. Furthermore, our institution reports BF data to the Joint Commission as required for hospital accreditation [5].

Per our hospital practice, any symptomatic infants were directly transferred from the delivery room to the NICU for further care. Following delivery, if the condition of the mother and her infant allowed, maternal-infant interactions such as holding, skin-to-skin contact, and BF were encouraged. Asymptomatic infants able to feed were transferred to the Newborn Nursery for routine care and glucose monitoring [7–9]. According to standard intrauterine growth charts, all infants were categorized as appropriate for gestational age (AGA), small for gestational age (SGA), large for gestational age (LGA) or macrosomic (birthweight ≥ 4000g) [23].

Screening for hypoglycemia (blood glucose < 40 mg/dl) was done via serial point of care testing (Accu-Chek®^)^ or by plasma glucose measurement in the laboratory (Beckman Coulter AU5800, Beckman Coulter Inc., Brea, CA, U.S.A.) starting within the first hour of life after the first feeding and every 2–4 hours thereafter as needed. Asymptomatic infants in the Newborn Nursery with hypoglycemia were promptly BF, formula fed (FF) or given dextrose gel and those with recurrent hypoglycemia were transferred to the NICU for further care. On admission to the NICU, most infants were started on intravenous (IV) dextrose and those who were able to feed were BF or FF [8].

BF was considered early if given within the first two postpartum hours. EBF was defined as direct feedings from the breast or expressed breast milk (EBM) alone or in combination with direct BF. Partial BF was defined as formula supplementation with direct BF or with EBM. BF was considered initiated if, during the 24 hours preceding hospital discharge, infants were EBF or BF partially [7–9]. Due to the retrospective study design, no follow-up information was available on infant feeding practices following hospital discharge.

## 3. Statistical analysis

Comparisons between women with Type 1 and Type 2 pregestational diabetes were made with Mood’s median tests for continuous variables and Chi square tests for categorical variables. Significance was established at a *p* value < 0.05. Univariate and multivariate logistic regression were used to ascertain the strength of association of Type 1 and Type 2 diabetes, obesity and GWG with EBF and BF initiation at discharge controlling for maternal variables (CHTN, preeclampsia, mothers age, race, health insurance type, smoking during pregnancy, BMI, parity, mode of delivery, prior BF, BF within two hours from birth) and neonatal variables (prematurity, AGA, SGA, LGA, admission to NICU, neonatal hypoglycemia and infant length of stay).

## 4. Results

### 4.1 Maternal and neonatal outcomes for women with diabetes and obesity

Clinical and demographic characteristics of 171 women with Type 1 and 294 women with Type 2 diabetes and obesity are shown in Table 1. Median pregestational BMI was lower for women with Type 1 (35 kg/m^2^) than for Type 2 (39 kg/m^2^). Among those with Type 1, obesity grade 1 (40 vs 26%) was higher while that of grade 3 was lower (29 vs 49%) than among women with Type 2 diabetes. Prevalence of obesity grade 2 (31 vs 25%), rates of CHTN alone (18 vs 32%) and preeclampsia superimposed on CHTN (7 vs 8%) were comparable for women with Type 1 and Type 2. Preeclampsia as a single co-morbidity was more common among Type 1 than Type 2 (20 vs 5%). Preeclampsia alone or superimposed on CHTN combined affected 47 (27%) women of Type 1 and 39 (13%) of Type 2 (*p ≤* 0.001). All women with preeclampsia with severe features received 24 hours of magnesium sulfate seizure prophylaxis.

**Table 1:** Maternal and Neonatal outcomes for women with diabetes and obesity.

	Type 1	Type 2	*p*
Mother-Infant Dyads no. (%)	171 (67)	294 (89)	0.0001
Chronic Hypertension no. (%)	30 (18)	70 (24)	NS
Chronic Hypertension with preeclampsia no. (%)	12 (7)	24 (8)	NS
Preeclampsia no. (%)	35 (20)	15 (5)	0.0001
Pregestational BMI kg/m^2^ median (IQR)	35 (32, 40)	39 (34, 45)	0.0001
Obese grade 1 BMI 30–34.9 kg/m^2^ no. (%)	69 (40)	76 (26)	0.001
Obese grade 2 BMI 35–39.9 kg/m^2^ no. (%)	53 (31)	73 (25)	NS
Obese grade 3 BMI ≥ 40 kg/m^2^ no. (%)	49 (29)	145 (49)	0.0001
Mothers age (y) median (IQR)	30 (25, 34)	33 (29, 37)	0.0001
Race			
White no. (%)	118 (69)	115 (39)	0.03
African American no. (%)	42 (25)	72 (24)	NS
Hispanic no. (%)	9 (5)	75 (26)	0.0001
Other no. (%)	2 (1)	32 (11)	0.0001
Public Assistance no. (%)	69 (40)	181 (62)	0.0001
Former smokers no. (%)	25 (15)	56 (19)	NS
Current smoker no. (%)	10 (6)	20 (7)	NS
Primiparous no. (%)	85 (50)	95 (32)	0.0001
Mode of Delivery			
Vaginal no. (%)	59 (35)	113 (38)	NS
Primary cesarean no. (%)	63 (37)	82 (28)	NS
Repeat cesarean no. (%)	49 (29)	99 (34)	NS
Mother length of stay (d) median (IQR)	4 (3, 5)	3 (3, 4)	0.0001
*Neonatal outcomes*			
Gestational age (w) median (IQR)	37 (36, 38)	38 (37, 39)	0.0006
Late preterm delivery no. (%)	54 (31)	49 (17)	0.0003
Birthweight (g) median (IQR)	3614 (3180, 3950)	3478 (3118, 3940)	0.04
Appropriate for gestational age no. (%)	93 (55)	170 (58)	NS
Large for gestational age no. (%)	74 (43)	114 (39)	NS
Small for gestational age no. (%)	4 (2)	10 (3)	NS
Macrosomia no. (%)	36 (21)	64 (22)	NS
Neonatal hypoglycemia no. (%)	101 (59)	123 (42)	0.0004
Admission to NICU no. (%)	77 (45)	69 (23)	0.0001
Infant length of stay (d) median (IQR)	3 (2, 4)	3 (2, 3)	0.001
*Infant feeding at discharge*			
Exclusive BF no. (%)	51(30)	72(24)	NS
Partial BF no. (%)	78(46)	132(45)	NS
Formula feeding no. (%)	42(25)	90(31)	NS
Breastfeeding Initiation no. (%)	129(75)	204(69)	NS

Women with Type 1 diabetes were more likely to be primiparous (50 vs 32%), younger (median age 30 vs 33), white (69 vs 39%) and less often Hispanic (5 vs 26%) than those in the Type 2 group. Smoking during pregnancy occurred with similar frequency among women with Type 1 and Type 2 diabetes (6 vs 7%). Public assistance was more common among women with Type 2 diabetes (62 vs 40%). Cesarean deliveries occurred with similar frequency (66 vs 62%) in both groups, while maternal length of hospital stay was slightly longer among Type 1 diabetics.

Infants born to women with Type 1 were of lower median gestational age (37 vs 38w) and higher median birthweight (3614 vs 3478g) than those of Type 2. Both groups were comparable in incidence of SGA (2 vs 3%), LGA (43 vs 39%)and AGA infants (55 vs 58%).

Neonatal hypoglycemia (59 vs 42%) and admission to the NICU (45 vs 23%) was more common among infants born to women in the Type 1 group. Considering the similarities in diagnoses, 77 infants from the Type 1 group and 69 infants from the Type 2 group admitted to the NICU were combined for analysis. Eighty-three of the 146 (57%) infants were admitted to the NICU directly from the delivery room while the remaining 63 (43%) stayed in the newborn nursery for a short time prior to transfer. Primary admission diagnoses to the NICU included hypoglycemia (50%), respiratory distress (27%), apnea-bradycardia-cyanosis (10%), temperature instability-hypotonia-poor feeding (10%) and miscellaneous (3%). EBF and BF initiation at discharge were similar for Type 1 and Type 2 groups (30 vs 24%) and (75 vs 69%), respectively. A comparison of BF outcomes for 146 infants admitted to the NICU and 319 others admitted to the Newborn Nursery showed that exclusive BF (27 vs 26%) and BF initiation (72 vs 71%) were not different. On the other hand, BF outcomes for 223 infants with hypoglycemia and 242 others without hypoglycemia showed that exclusive BF rates were lower (20 vs 33%, *p* 0.003) while BF initiation (70 vs 73%) was no different between the two groups. All mothers and their infants were discharged home in good condition.

### 4.2 Study population according to infant feeding preference

Considering that our regression analysis showed that BF initiation (any BF) at discharge was more likely among women with Type 1 who intended to exclusively BF, we divided the entire population into 384 women who intended to exclusively BF and 81 who intended to partially BF (Table 2). Our data showed that intention to exclusively BF was more common among primiparous white women with Type 1 diabetes. Smoking during pregnancy occurred with similar frequency among women who intended exclusive BF and those who intended partial BF (5 vs 12%). Public assistance was more frequent among women who intended partial BF (77 vs 56%). At the time of discharge, EBF and BF initiation rates were higher among women who intended to BF exclusively compared to those who intended to BF partially (31 vs 6% and 77 vs 44%, respectively).

**Table 2:** Study population according to infant feeding preference.

	Intended EBF	Intended Partial BF	*p*
Mother-Infant Dyads no.	384	81	
Type 1 no. (%)	153 (40)	18 (22)	0.003
Type 2 no. (%)	231 (60)	63 (78)	0.003
Chronic Hypertension no. (%)	80 (21)	20 (25)	NS
Preeclampsia no. (%)	72 (19)	15 (19)	NS
Pregestational BMI kg/m^2^ median (IQR)	38 (34, 43)	36 (33, 43)	NS
Pregestational weight (kg) median (IQR)	90 (77, 111)	89 (75, 104)	NS
Gestational weight gain (kg) median (IQR)	12 (7, 20)	11 (6, 18)	NS
Mothers age (y) median (IQR)	31 (27, 36)	32 (28, 37)	NS
Race			
White no. (%)	210 (55)	23 (28)	0.0001
African American no. (%)	94 (24)	20 (25)	NS
Hispanic no. (%)	54 (14)	30 (37)	0.0001
Other no. (%)	26 (7)	8 (10)	NS
Public Assistance no. (%)	214 (56)	62 (77)	0.0005
Former smokers no. (%)	69 (18)	12 (15)	NS
Current smokers no. (%)	20 (5)	10 (12)	NS
Primiparous no. (%)	167 (43)	15 (19)	0.0001
Mode of Delivery			
Vaginal delivery no. (%)	137 (36)	35 (43)	NS
Primary cesarean no. (%)	133 (35)	12 (15)	0.0003
Repeat cesarean no. (%)	114 (30)	34 (42)	0.04
Prior breastfeeding experience no. (%)	168 (44)	43 (52)	NS
*Neonatal outcomes*			
Gestational age (w) median (IQR)	38 (37, 39)	37 (37, 38)	NS
Late preterm delivery no. (%)	86 (22)	17 (21)	NS
Birthweight (g) median (IQR)	3479 (3119, 3115)	3640 (3268, 4010)	0.02
Appropriate for gestational age no. (%)	227 (59)	36 (44)	0.02
Large for gestational age no. (%)	146 (38)	42 (52)	0.02
Small for gestational age no. (%)	11 (3)	3 (4)	NS
Macrosomia no. (%)	81 (21)	22 (27)	NS
Neonatal hypoglycemia no. (%)	190 (49)	34 (42)	NS
Admission to NICU no. (%)	127 (33)	19 (23)	NS
Infant length of stay (d) median (IQR)	3 (2,4)	3 (2,4)	NS
*Infant feeding at discharge*			
Exclusive breastfeeding no. (%)	119 (31)	5 (6)	0.0001
Partial breastfeeding no. (%)	178 (46)	31 (38)	NS
Formula feeding no. (%)	87 (23)	45 (56)	0.0001
Breastfeeding Initiation no. (%)	297 (77)	36 (44)	0.0001

*Macrosomia is defined by a birthweight ≥ 4000g.

### 4.3 Maternal and neonatal outcomes according to GWG

In order to calculate GWG, pregestational weight was measured in 76% and recalled in 24% of women with Type 1 and measured in 69% and recalled in 31% of women with Type 2 diabetes. Combining the entire study population of women with Type 1 and Type 2, we determined that 17 (4%) had *inadequate* GWG, 83 (18%) had a*dequate* GWG and 365 (78%) had e*xcessive* GWG. The 365 women with e*xcessive G*WG were compared to the 100 women with non-e*xcessive* GWG (Table 3). Those with e*xcessive* GWG were more likely to be Type 1, white, primiparous, delivered by primary cesarean and have lower pregestational weight. Smoking during pregnancy occurred with similar frequency among both groups of women (5 vs 12%). Public assistance was less common among women with *excessive* GWG (57 vs 69%). Birthweight and rate of LGA were higher among infants born to women with e*xcessive* GWG, however, the rates of hypoglycemia and the need for NICU care were similar to those in the non-e*xcessive* GWG group. At the time of discharge, the e*xcessive* and non*-excessive* GWG groups had similar EBF (27 vs 25%), partial BF (44 vs 47%) and any BF (72 vs 72%) rates. Two hundred and thirteen women of the 365 (58%) with e*xcessive* GWG were multiparous. One hundred and fifty-nine (75%) of them had prior BF experience. At the time of discharge, 30% of the multiparous with prior BF experience and 19% of the 67 without prior experience had EBF (*p* 0.007).

**Table 3:** Maternal and neonatal outcomes according to gestational weight gain.

	Excessive	Non-Excessive	*p*
Mother-Infant Dyads no. (%)	365 (78)	100 (22)	
Type 1 no. (%)	143 (39)	28 (26)	0.05
Type 2 no. (%)	222 (61)	72 (74)	0.05
Chronic Hypertension no. (%)	73 (20)	27 (27)	NS
Preeclampsia no. (%)	74 (20)	13 (13)	NS
Pregestational BMI kg/m^2^ median (IQR)	37 (34, 43)	38 (33, 44)	NS
Pregestational weight (kg) median (IQR)	86 (74, 104)	100 (87, 120)	0.0001
Gestational weight gain (kg) median (IQR)	15 (10, 21)	1 (0, 4)	0.0001
Mothers age (y) median (IQR)	32 (27, 36)	31 (28, 36)	NS
Race			
White no. (%)	194 (53)	39 (39)	0.01
African American no. (%)	88 (24)	26 (26)	NS
Hispanic no. (%)	58 (16)	26 (26)	NS
Other no. (%)	25 (7)	9 (9)	NS
Public Assistance no. (%)	207 (57)	69 (69)	0.03
Former smokers no. (%)	66 (35)	15 (15)	NS
Current smokers no. (%)	18 (5)	12 (12)	NS
Primiparous no. (%)	152 (42)	30 (30)	0.04
Mode of Delivery			
Vaginal delivery no. (%)	127 (35)	45 (45)	NS
Primary cesarean no. (%)	122 (33)	23 (23)	0.05
Repeat cesarean no. (%)	116 (32)	32 (32)	NS
Prior breastfeeding experience no. (%)	159 (44)	47 (47)	NS
*Neonatal outcomes*			
Gestational age (w) median (IQR)	38 (37, 38)	38 (37, 39)	NS
Late preterm delivery no. (%)	83 (23)	20 (20)	NS
Birthweight (g) median (IQR)	3570 (3203, 3970)	3325 (3002, 3880)	0.004
Appropriate for gestational age no. (%)	195 (53)	68 (68)	0.01
Large for gestational age no. (%)	160 (44)	28 (28)	0.004
Small for gestational age no. (%)	10 (3)	4 (4)	NS
Macrosomia no. (%)	85 (23)	18 (18)	NS
Neonatal hypoglycemia no. (%)	180 (49)	44 (44)	NS
Admission to NICU no. (%)	115 (32)	31 (31)	NS
Infant length of stay (d) median (IQR)	3 (2, 4)	3 (2, 4)	NS
*Infant feeding at discharge*			
Exclusive breastfeeding no. (%)	99 (27)	25 (25)	NS
Partial breastfeeding no. (%)	162 (44)	47 (47)	NS
Formula feeding no. (%)	104 (28)	28 (28)	NS
Breastfeeding Initiation no. (%)	261 (72)	72 (72)	NS

### 4.4 Maternal

#### Maternal obesity, GWG and infant feeding at discharge

The study population was categorized according to obesity grades (Table 4). Thirty-one percent of women were classified as grade 1, 27% grade 2 and 42% were grade 3. Prevalence of *adequate* GWG increased from grade 1 to grade 3 (12–24% *p <* 0.005), while *inadequate* GWG was found only in Grade 1 (12%). The prevalence of *excessive G*WG among the three obesity groups was similar.

**Table 4:** Maternal obesity, gestational weight gain and infant feeding at discharge.

		Gestational Weight Gain			Breastfeeding at Discharge		
Obesity	No. (%)	*Adequate*	*Inadequate*	*Excessive*	Exclusive	Partial	Initiated
Grade 1	145 (31)	17 (12)	17 (12)	111 (77)	40 (28)	69 (48)	109 (76)
Grade 2	126 (27)	20 (16)	0 (0)	106 (84)	42 (33)	50 (40)	92 (73)
Grade 3	194 (42)	46 (24)	0 (0)	148 (76)	42 (22)	90 (46)	132 (68)
All grades	465	83 (18)	17 (4)	365 (78)	124 (27)	209 (45)	333 (72)

Obesity (BMI): Grade 1 (30–34.9 kg/m^2^), Grade 2 (35–39.9 kg/m^2^), Grade 3 (≥ 40 kg/m^2^).

At the time of discharge, the rate of EBF was higher among grades 1 and 2 (28 and 33%) than in grade 3 (22%) (*p* 0.03). Partial BF occurred with similar frequency across the three obesity groups (48, 40 and 46%, respectively). Consequently, BF initiation was comparable between grade 1 and grade 2 (76 vs 73%) but slightly lower among grade 3 (68%). Of all 465 study patients combined, there were 124 (27%) who EBF and of them 77 (62%) BF by direct BF while 47 (38%) received exclusive EBM. During the hospitalization, 12 infants from the EBF group received donor human milk.

### 4.5 Regression analysis and GWG associations

Our regression analysis showed that the stronger predictors for EBF at discharge were intention to BF exclusively for women with Type 1 DM (a OR 9.580, 95% CI, 1.163–78.900) and for women with Type 2 DM (a OR 6.571, 95% CI, 2.240–19.279). Conversely, the stronger predictors of failure for EBF were neonatal hypoglycemia for Type 1 DM (a OR 0.317, 95% CI, 0.154–0.651) and for Type 2 (a OR 0.419, 95% CI, 0.230–0.765).

The stronger predictors of BF initiation (any BF) at discharge for Type 1 DM were intention to BF exclusively (a OR 4.598, 95% CI, 1.419–14.904) and for Type 2 DM (a OR 5.050, 95% CI, 2.540–10.042). Conversely, the major obstacles for BF initiation (any BF) failure at discharge for Type 2 were preeclampsia (a OR 0.294, 95% CI, 0.126–0.684), public assistance (a OR 0.390, 95% CI, 0.195–0.782) and neonatal hypoglycemia (a OR 0.547, 95% CI, 0.305–0.982).

After controlling for potential confounders, none of the GWG categories (*adequate*, *inadequate* and *excessive)* were predictors of EBF or BF initiation at discharge.

## 5. Discussion

The American Academy of Pediatrics and the Academy of Breastfeeding Medicine recommend EBF for all healthy infants during birth hospitalization and beyond [6,24]. These organizations acknowledge that in the presence of maternal and neonatal morbidities, other nutritional options may be needed to temporarily replace or supplement BF [6,24]. Concurrently, since January 2014, the Joint Commission mandated the reporting of EBF rates during hospitalization for healthy infants to retain their maternity accreditation [5]. In a recent study of maternity care practices and policies in 1,305 hospitals in the United States, the mean in-hospital EBF rate for infants in the general population was 51.4% [6]. Recently, we reported EBF rates in women with mild CHTN (47%) and with severe CHTN (50%) [25], with preeclampsia (39%) and with severe preeclampsia (37%) [26], with preeclampsia superimposed on pregestational diabetes (18%) [27], with CHTN superimposed on pregestational diabetes (19%) [28], and with pregestational diabetes with prior BF experience (33%) and without prior BF experience (11%) [9]. In line with the above, the low EBF rates associated with e*xcessive* GWG for Type 1 and Type 2 (27%), prior BF experience (33%), Grade 1 obesity (28%), Grade 2 (33%) and Grade 3 (22%) reported here are similar to those of women with pregestational diabetes and superimposed comorbidities described above [9,25–28]. Based on those experiences we are inclined to speculate that the high incidence of hypoglycemia and NICU admission unique to pregestational diabetes could explain the further decrease on the rate of EBF. In addition, women who BF exclusively by EBM without any direct BF is also of concern because pumping without feeding at the breast is associated with shorter BF duration [29].

Delayed lactogenesis II may be a contributing factor to the low EBF among women with diabetes, obesity and *excessive* GWG reported here [11,30,31]. Obesity is also likely to be associated with comorbidities such as preeclampsia, CHTN and preexisting diabetes that increase the risk for cesarean delivery, indicated prematurity and neonatal hypoglycemia, factors known to negatively influence BF rates [26,32,33]. GWG above the target recommendations has also been associated with the development of childhood obesity [34]. Given that EBF has been shown to lower the risk for childhood obesity by up to 25% [34], improvement in BF rates in this population is a desirable objective.

Our data also showed that women with *excessive* GWG and no prior BF experience had the lowest EBF at discharge. A positive BF experience improves attitude, confidence, self-efficacy, motivation and intention to BF [9,34]. Negative BF experiences are related to maternal or neonatal morbidities, to difficulties inherent to lactation such as perception of low milk supply, suck or latch problems, mastitis or nipple fissures [9,35,36]. A detailed BF history can provide insight into obstacles that led to unsuccessful BF and may help to define appropriate preventive or corrective strategies for subsequent pregnancies [9].

In the present study we observed BF initiation rates for women with Type 1 (76%), with Type 2 (69%), with Grade 1 obesity (76%), Grade 2 (73%) and Grade 3 (68%). Considering the above, the BF initiation rates reported here, albeit lower, are comparable to the 83.2% reported recently for the general U.S. population [37]. Obstacles known to associate with low EBF and BF initiation affecting our study population included lack of prior BF experience, preeclampsia, CHTN, pregestational diabetes, obesity, complications of labor, cesarean delivery, premature birth, excessive GWG, neonatal hypoglycemia, admission to NICU, late BF, formula supplementation, delayed lactogenesis II and maternal-infant separation [7,25–30,38]. The low current smoking rate observed in all subgroups of women studied here may be due to the success of prenatal smoking cessation programs [39].

The high rate of partial BF at the time of discharge may characterize two groups of women. Those who intended only to BF and were unable to EBF at discharge may be a group of women who with support and encouragement could reinitiate and maintain EBF. The other group was composed of women who prenatally intended partial BF and accomplished their goal. Unfortunately, our data also showed that one half of women who intended partial BF failed and were discharged feeding formula only, a circumstance unlikely to be reversed [38]. In line with previous reports, our data showed that public assistance was more common among women who intended partial BF at discharge and for those women with type 2 diabetes [40,41]. It is evident that each of these groups might require different educational strategies if their lactation goals are to be achieved.

The reported prevalence of *excessive* GWG ranged from 36 to 73% for women with obesity [15–16,42–45], from 36 to 54% for women with gestational diabetes [17,45,46] and from 41 to 64% for women with obesity and pregestational diabetes [46,47]. The higher prevalence of *excessive* GWG (76%) reported here for the three obesity grades may be the result of the combination of diabetes mellitus and obesity that characterized our study population.

In 2012, Bartok et al, using the 2009 guidelines, noted that neither BMI nor GWG was associated with BF outcomes [14]. Subsequently, Winkvist et al reported that Norwegian women with obesity who experience *excessive* GWG had a higher inability to sustain full or any BF [15]. More recently, it was reported that among Brazilian women with obesity, *excessive* GWG was not associated with any EBF or BF [16]. However, Haile et al reported that among women with gestational diabetes and *excessive* GWG, the odds of EBF during the neonatal period and at three months postpartum were lower compared to women without diabetes [17]. In our special population of women with multiple co-morbidities, *excessive* GWG may have been a contributor, but it was not an independent predictor of low EBF or BF initiation.

Our regression analysis showed that neonatal hypoglycemia was a predictor of EBF failure among women with Type 1 and Type 2 and obesity. Indeed, hypoglycemia, lack of early BF and admission to the NICU are well known interrelated obstacles to EBF and BF initiation, especially among infants born to women with gestational and pregestational diabetes [8,26,27,38]. Early BF or early FF may facilitate glycemic stability and prevent or correct hypoglycemia avoiding the need for dextrose gel, IV treatment or formula supplementation [8]. However, our data showed that approximately one third of the infants with hypoglycemia required admission to the NICU with the unavoidable mother-infant separation that is another strong physical and psychological barrier to EBF.

It is known that racial discrepancies are accompanied by low rates of any BF and EBF in African Americans and Hispanics as compared to non-Hispanic whites [48]. In line with the above, our data on women with Type 2 diabetes showed that BF initiation among Hispanics and African Americans was lower than among those of non-Hispanic white women. However, rates of excessive GWG were comparable across ethnic groups.

A limitation of this investigation is that the calculation of GWG was based on pregestational weight measured during the first prenatal visit or by maternal recall at the time of delivery. Another limitation is the lack of follow-up information regarding infant feeding choices after discharge from the hospital. The strength of this study rests on the use, for the first time, of GWG guidelines applicable to women with various degrees of obesity. Finally, the data of the obstetrical and neonatal population was obtained directly from medical records, and not via post-delivery maternal questionnaires.

In conclusion, the prevalence of *excessive* GWG is high among women with diabetes, but similar across all grades of obesity. Compared to healthier maternal populations, women with Type 1 and Type 2 diabetes, obesity and *excessive* GWG are likely to have very low rates of EBF and similar BF initiation rates. Women who intended to BF only are more likely to EBF while those who intended partial BF are at higher risk of BF initiation failure. Diverse maternal and neonatal comorbidities create obstacles to lactation, however, *excessive* GWG is not associated with either EBF success or failure among women with pregestational diabetes and obesity.
